# From Spontaneous Ignitions to Sensorimotor Cell Assemblies via Dopamine: A Spiking Neurocomputational Model of Infants’ Hand Action Acquisition

**DOI:** 10.3390/brainsci16020158

**Published:** 2026-01-29

**Authors:** Nick Griffin, Andrea Mattera, Gianluca Baldassarre, Max Garagnani

**Affiliations:** 1 Department of Computing, Goldsmiths, University of London, London SE14 6NW, UK; 2 Institute of Cognitive Sciences and Technology, National Research Council, 00196 Rome, Italy; andrea.mattera@istc.cnr.it (A.M.); gianluca.baldassarre@istc.cnr.it (G.B.); 3 Department of Philosophy, Alma Mater Studiorum—University of Bologna, 40126 Bologna, Italy; 4 Brain Language Laboratory, Department of Philosophy and Humanities, Freie Universität Berlin, 14195 Berlin, Germany

**Keywords:** reinforcement learning, dopamine, spontaneous action, neurobiologically realistic modeling, deep network, cell assembly

## Abstract

**Background/Objectives**: From birth, infants learn how to interact with the world through exploration. It has been proposed that this early learning phase is driven by motor babbling: the spontaneous generation of exploratory movements that are progressively consolidated through associative mechanisms. This process leads to the acquisition of a repertoire of hand movements such as single- or multi-finger flexion, extension, touching, and pushing. Later, in a second phase, some of these movements (e.g., those that happen to enable access to biologically salient stimuli, such as grasping food) are further reinforced and consolidated through rewards obtained from the environment. However, the neural mechanisms underlying these processes remain unclear. Here, we used a fully neuroanatomically and neurophysiologically constrained neural network model to investigate the brain correlates of these processes. **Methods**: The model consists of six neural maps simulating six human brain areas, including three pre-central (motor-related) and three post-central (sensory-related) regions. Each map is composed of excitatory and inhibitory spiking neurons, with biologically constrained within- and between-area connectivity forming recurrent circuits. Hand action execution and corresponding haptic perception are simulated simply as activity in primary motor and somatosensory model areas, respectively. During an initial “exploratory” phase, the network learned, via Hebbian mechanisms, associations—as emerging distributed cell assembly (CA) circuits—linking “motor” to corresponding “haptic feedback” patterns. As a result of this initial training, the model began to exhibit spontaneous ignitions of these CA circuits, an emergent phenomenon taken to represent internally generated, non-stimulus-driven attempts at hand action exploitation. In a second phase, a global reward signal, simulating dopamine-mediated reward encoding, was applied to only a subset of “successful” actions upon their noise-driven ignition. **Results**: During the first exploratory phase, the neural architecture autonomously developed “action-perception” circuits corresponding to multiple possible hand actions. During the subsequent exploitation phase, positively reinforced circuits increased in size and, consequently, in frequency of spontaneous ignition, when compared to non-rewarded “actions”. **Conclusions**: These results provide a mechanistic account, at the cortical-circuit level, of the early acquisition of hand actions, of their subsequent consolidation, and of the spontaneous transition of an agent’s behavior from exploration to reward-seeking, as typically observed in humans and animals during development.

## 1. Introduction

From the moment when we are first born, we start to learn how to interact with the world around us, behaving in an exploratory or curiosity-driven manner [1,2,3,4]. Curiosity can guide our attention toward unexpected events [5,6], and our exploratory behavior includes spontaneous self-touching that develops from closed fists to self-grasping [7,8], eventually leading to learning how to explore objects with our hands [9,10]. Later, some acquired actions can lead to attaining biologically salient outcomes perceived as rewarding, thus strengthening and consolidating them as “successful” [11,12]. However, the neural mechanisms that drive the spontaneous emergence of actions during the initial exploratory phase, and their later exploitation leading to the endogenous selection and consolidation of useful actions, remain largely unknown.

Here, we deployed a large-scale, deep, neuroanatomically grounded neurocomputational model with spiking neurons to investigate the brain mechanisms underlying the initial, random-like generation (or exploratory), and later selective, and consolidating (or exploitative) phases that, together, constitute the spontaneous process of skilled hand action learning in infants (the term “deep” is used here simply to indicate that the network consists of a hierarchy of multiple reciprocally connected areas, with no reference to the standard sense of this term used in machine learning or AI today). We note that we only modeled the cortical areas and neural mechanisms implicated in these processes and did not simulate the execution of actions in terms of muscle contractions or the effects of these on an environment.

The use of large-scale neurocomputational models replicating the structure and function of the human brain appears to be a promising direction in the development of neuromorphic artificial intelligence (AI) systems. Here, we built on computational theories of motor babbling, themselves derived from the extensive empirical and modeling literature of vocal babbling. More specifically, in a number of previous simulation studies, the present neural architecture was applied to model the early exploratory stages of speech learning [13,14,15,16,17,18,19,20,21,22,23] (see Pulvermüller [24] for a recent review). During vocal babbling, in the early developmental phases of speech acquisition [25], near-simultaneous correlated activity is known to be present in different brain parts, especially those areas controlling speech output (left inferior prefrontal cortex) and those where neurons respond to auditory features of speech (left superior temporal lobe). These areas are connected via long-distance white matter fiber tracts (see Pulvermüller [26], or any of the above publications, for the supporting evidence). Our hypothesis there, as here, was that, through Hebbian learning mechanisms [27], such connections allow for the acquisition of sensory–motor associations between co-occurring cortical patterns of activity. In the case of vocal babbling, listening to speech sounds involving specific articulators leads to the “lighting up” of the corresponding motor representations (and vice versa). A significant body of experimental evidence indeed confirms the presence of speech–motor associations as networks of strongly interconnected neurons distributed between left superior temporal and inferior frontal cortex and their role in language processing [26,28,29,30,31,32,33,34] (see Pulvermüller and Fadiga [35] for a review).

Motor babbling draws inspiration from evidence, theories, and models initially developed for vocal babbling, although the empirical and computational bases supporting it are comparatively less robust. Despite this, motor babbling is widely believed to contribute substantially to early motor development in infants. It relies on a basic learning mechanism in which infants generate their own training data through the production of spontaneous, random movements [36]. Learning systems grounded in motor babbling typically employ associative processes, although more advanced forms of learning, such as reinforcement learning, can be used and use some degree of random movement generation to discover novel behaviors. While we have assumed here that motor babbling is sufficient for an infant to produce their own training data, such random sampling may not be enough or efficient in the real world. For example, as extensively reviewed in Adolph and Hoch [37], infant bodies undergo numerous physical changes that affect motor behavior, which may require adaptation of existing actions, and the action space is more continuous where multiple different muscle contractions can produce similar behavioral outcomes [37].

Numerous biologically inspired models within developmental robotics have been introduced to emulate motor babbling [38,39,40,41,42,43,44]. These models converge on the principle that exploratory movements enable the establishment of associations between the internal representations of movements and those of their sensory consequences. When these sensory effects later become desirable, activating their representations can reactivate the motor patterns that originally produced them. Kuperstein’s pioneering study [38] showed that the execution of random movements allows a computational eye–arm system to link perceived object-in-hand locations with corresponding arm configurations, knowledge that can subsequently be used to perform reaching tasks. Caligiore et al. [40] demonstrated that embedding biological constraints—such as leaky neurons, equilibrium-point muscle models, population coding, and Hebbian learning—within motor-babbling neural networks yields emergent properties, including Mexican-hat-shaped lateral connectivity and bell-shaped velocity profiles reminiscent of human movements.

Using the Babybot humanoid platform, Natale et al. [45,46] investigated the acquisition of reaching and grasping via motor babbling. The robot first learned to visually fixate its own hand as motor babbling drove it to various spatial locations. After establishing associations between gaze direction and arm posture, Babybot was able to reach toward external objects by combining a pre-programmed object-recognition system to orient gaze with the previously learned reaching behavior to guide the hand. Extending this line of work, Caligiore et al. [41] demonstrated that motor babbling combined with associative learning can support the emergence of more complex behaviors when augmented with additional mechanisms. For instance, co-ordinated reaching and grasping—requiring a sequence of ordered movements—can be produced by incorporating hand-closure reflexes akin to those of newborn infants. Within a study linking motor babbling and reinforcement learning, Caligiore et al. [47] introduced a neural model that used such a learning paradigm to control a muscle-like system, reproducing key kinematic and dynamic characteristics of human reaching development.

Despite these advances, previous models share common limitations: they rely on abstract neural architectures and treat associative and reinforcement learning as independent and disconnected processes. In the present work, we addressed these shortcomings by introducing a biologically grounded model that unifies Hebbian and reinforcement learning within a single integrated framework.

In our previous simulation studies of vocal babbling, the process of Hebbian association between sensory and motor patterns was modeled through repeated simultaneous activation of pre-determined sets of cells in the model equivalent of primary auditory (A1) and primary motor (M1) cortices. The presence of an activity pattern in the latter was taken to represent the spontaneous motor-cortical activity that one might observe in M1 during the babbling phase [25]; the pattern presented as input to the former simulated the cortical activation that would result in A1 from the near-simultaneous perception of the speech sounds generated by the articulatory movements driven by the activity in M1. An analogous approach was adopted here, except that, instead of simulating vocal babbling, we simulated motor babbling. Accordingly, instead of modeling six speech-related perisylvian areas (see Garagnani et al. [13], their Figure 3), we modeled six frontal and parietal areas implicated in hand/grasping action (see Section 2.2 for details).

Dopamine, broadly recognized to signal if a reward or stimulus is predicted or not [11,48,49,50], has been suggested as a means to reinforce relevant behaviors [51,52,53] in a similar way to the error signal of temporal difference learning [50,54,55]. Recently, Kasdin et al. [56] showed that dopamine mediates the internally guided trial-and-error learning of songs in zebra finches as an example of its involvement in the development and reinforcement of natural behaviors. Within other categories of learning, dopamine has been shown to have a causal role in cue-reward associative learning and also in extinction learning [57,58]. More specifically, dopamine has been found to play a role in long-term potentiation (LTP) and long-term depression (LTD) in the PFC, displaying an inverted-U-shaped effect [59,60,61,62]. This role in LTP/LTD has been demonstrated by a number of studies in humans [63], primates [64,65], and rodents [66,67,68,69,70,71,72,73], along with several good collective reviews [74,75,76,77]. Additionally, there is abundant evidence that dopamine modulates working memory (WM), displaying inverted-U-shaped effects analogous to those seen with LTP/LTD [78,79].

The above body of experimental works has led to a surge of computational studies investigating the role of reward signals as a third factor in brain-based modeling of synaptic plasticity—see, e.g., useful reviews in the contexts of the exploration–exploitation balance [80], spike-timing-dependent plasticity [81,82], and the sensory cortices [83]—giving rise to a rapidly emerging area known as neo-Hebbian learning [84]. Of particular relevance here are the works by Sheynikhovich and colleagues, who developed a learning rule where the LTP/LTD threshold and amplitude of plasticity were modulated by dopamine [85,86], which reproduced a number of the previously mentioned experimental results regarding the inverted-U-shaped effect of dopamine in the PFC [59,60,61,62]. Simulation studies have also replicated the mentioned modulatory effects of dopamine on working memory [87,88]. These seminal studies have been very important for improving our understanding of dopamine’s modulatory role at the level of neurons and their synapses; however, the exact effects of this modulation at the population and cortical-circuit level, and the link between these and macroscopic behavior, remain unclear.

In line with the above, the present investigation proceeded from two main assumptions, namely, that (H1) infants spontaneously explore their peri-personal space through motor babbling (hand and finger) actions and that (H2) dopamine plays a role in reinforcing some of these actions (e.g., those that happen to result in a successful object grasp producing reward). Accordingly, we ask here (i) which neural processes may underlie the initial phase of hand action acquisition, during which the infant randomly explores a rich variety of possible finger and/or hand movement combinations; (ii) which brain mechanisms relying on reward may mediate the evaluation of such actions’ outcomes; and (iii) which cortical processes might drive the transition of the infant’s behavior from one of purely random exploration to one of “exploitation” in which most actions executed are “successful” or skilled ones. The latter two points can be seen as addressing the more general question about which neural mechanisms may lead an animal to spontaneously develop a reward-seeking behavior. Finally, we also ask whether (iv) dopamine-mediated reward (known to affect working memory) may be playing a facilitatory role in the storage and consolidation of the set of actions emerging as most relevant or successful by enabling their longer maintenance in short-term memory.

To address these questions and shed light on the underlying cortical processes, we built upon and extended an existing brain-constrained model of frontotemporal cortical areas, successfully used in the past to mechanistically explain, at the cortical-circuit level, behavioral indexes, as well as the emergence, dynamic topographies, and slow ramping, of neural activity underlying volitional action decisions in the human brain [15,89]. Brain-constrained architectures incorporate constraints taken directly from well-documented neuroanatomical and neurophysiological features of the mammalian cortex—see Section 2, “Materials and Methods”, below, for more details.

### Elaboration of the Hypothesis

In several studies, the emergence of endogenous hand (and, similarly, speech) action decisions in the cortex is explained on the basis of spontaneous, noise-driven dynamics of associative “action-perception” circuits—a.k.a. “cell assembly” circuits [27,90,91,92], CAs for short—distributed sets of strongly and reciprocally connected neuronal cells that emerge across the network as a result of Hebbian-like learning. Unlike in similar previous works (which simulated the formation of *visuo*-motor CA associations as induced by repeated hand action in the presence of a specific visual stimulus), however, here we modeled the emergence of action-perception circuits linking motor with correlated *haptic* activity patterns, putatively co-occurring in primary motor and somatosensory cortices during spontaneous exploratory finger/hand action (in the presence of an object). This requires the network to model a different set of cortical regions, specifically including areas in the parietal lobe, and their interconnections (see the Methods section below for details). A second significant novel element of the present investigation consists of the implementation of a learning mechanism that simulated dopamine-modulated synaptic plasticity, grounded in neurophysiological evidence; this allowed us to investigate the effects of reward on spontaneous decisions to act, an aspect entirely absent in any of the previous studies that used the present architecture. Finally, here, we also improve on the neurobiological realism of the neurocomputational model by using spiking artificial neurons instead of graded-response ones.

Our working hypotheses (relating directly to H1 and H2, mentioned earlier) were that, while the initial exploratory phase of skilled hand action learning may be driven by internal (neuronal) noise, the agent/infant gradually develops a reward-seeking behavior as the combined result of (a) the dynamics of cortical action-perception circuits, which allegedly exhibit noise-driven spontaneous reactivation (also known as “ignition”), and of (b) the reinforcement signal, which rewards only a subset of the initial CA repertoire. This hypothesis builds upon the results of previous simulations with the same neural architecture, showing that the presence of uniform white noise in the network (simulating baseline neuronal firing) is sufficient to induce periodic spontaneous ignitions of the learned associative CA circuits in seemingly random order [15,89,93]. Importantly, these results revealed no *bias* in the probability of CA ignition other than CA size (measured as the number of cells forming the circuit). Hence, if CA size is controlled for, the different CA circuits (together modeling a repertoire of possible finger/hand actions, for the same or different objects) tend to exhibit the same probability of ignition (measured as frequency of spontaneous ignitions over time). Taking—in line with the above results—the spontaneous ignition of an action-perception circuit to represent the model correlate of an internally generated, non-stimulus-driven decision to execute a hand action, a situation in which all CAs have equal likelihood of ignition (i.e., all actions are spontaneously attempted, in random order) replicates the behavior characteristic of an initial, random-like exploratory phase. Accordingly, we expect the simulation results to reveal no differences in the spontaneous ignition frequencies of different CAs having equal size.

Our second, and crucial, hypothesis was that rewarded CA circuits should gradually grow larger than non-rewarded ones due to the presence of a global reward signal enhancing learning everywhere in the network. This situation modeled a scenario in which only some of the possible hand actions result in a successful object grasp producing reward. These “successful” CA circuits, having grown larger than the “unsuccessful” ones, would, therefore, be more likely to spontaneously ignite again in the future. In sum, the net effect of introducing a reward mechanism in the model should be that of driving the system to gradually acquire a *bias* toward the spontaneous execution of successful (rewarded) “hand action” CA circuits over other (non-rewarded) ones. We expected to observe this in the simulation results as a significant difference in both average size and probability of spontaneous ignition between rewarded and non-rewarded CA circuits. Macroscopically, such behavior would mimic that of an agent exhibiting a natural tendency to transition from an initial, random-driven exploration to a reward-seeking phase, as typically observed in humans and other animals during development [94].

## 2. Materials and Methods

**Figure 1:**
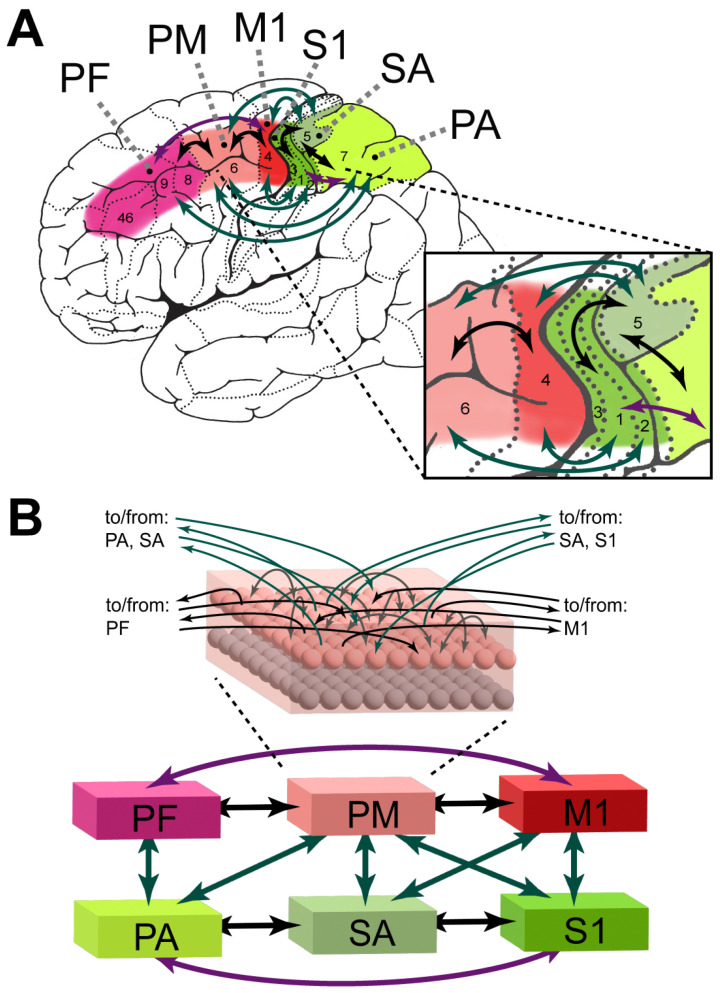
Simulated brain areas, model architecture, and the mapping between them. (**A**) Cortical areas modeled. These included three areas in dorsolateral prefrontal (Brodmann Areas, BA, 8/9/46), premotor (BA 6), and primary motor (BA 4) cortices, responsible for hand/finger motor actions, and three in the parietal cortex, the primary somatosensory (BA 3/1/2), somatosensory association (BA 5), and posterior parietal association (BA 7) cortices, known to be involved in perception of haptic and proprioception information coming from the hand and fingers. The inset depicts the four main “central”—taking the central sulcus as a symmetry axis—areas, PM, M1, S1, and SA, and their documented anatomical connections (black, green, and purple arrows). (**B**) Model areas and between-area connections implemented. (**Inset**) The PM area is enlarged to illustrate internal area structure (consisting of two layers of spiking excitatory and inhibitory cells), showing within- (gray) and between-area (green and black arrows) links. Reciprocal connections between the two layers are not shown. The color coding indicates the mapping between the model and corresponding brain areas (and white matter fiber tracts) it simulates. Note the 1-1 correspondence between neuroanatomical links known to exist between the modeled brain areas (arrows in panel (**A**)) and the inter-area projections implemented in the model (arrows in (**B**))—in particular, no link between two model areas was implemented unless extant experimental evidence indicated the presence of white matter fiber tracts connecting the corresponding brain regions. Black-, purple-, and green-colored arrows indicate, respectively, the presence of a well-documented direct synaptic link between (i) cortically adjacent areas, (ii) non-adjacent areas belonging to the same (motor or somatosensory) system (“jumping” links), and (iii) non-adjacent cortical areas located in two different systems (long-distance cortico-cortical connections). See the main text for details. *Panel (**A**) is adapted from* [15].

We used a brain-constrained architecture to investigate the reward-driven acquisition of new hand/finger motor skills, such as object grasping and tool use. In general, neuroanatomical and neurobiological constraints can be taken from different levels of brain organization [95]. For example, if each model area represents a well-identified region of the cortex, one may enforce—as we did here—that links between such areas may be added to the model *only* if white matter tracts between corresponding regions are known to exist. In addition, in a fully constrained model, any implemented mechanism must mimic a neurophysiological process well known to occur in the mammalian cortex (action potentials, spontaneous baseline firing, long-term potentiation, etc.). Accordingly, the following brain constraints were implemented here:

- Neurophysiological dynamics of single cells, including transformation of membrane potentials into neuronal outputs (spikes), temporal summation of inputs, and adaptation, were implemented [96,97], following Garagnani et al. [13,98];
- Synaptic weights were modified through Hebbian-like long-term potentiation (LTP) and long-term depression (LTD) [99], following Garagnani et al. [13,98];
- LTP and LTD mechanisms were modulated by a simulated dopamine signal, closely following known neurophysiological data about reward-modulated learning processes in the mammalian cortex—see Section 2.2.1 and Section 2.3 for details;
- Area-specific and local inhibition-implemented global and local activity regulation [91,100];
- Six areas known to be implicated in hand/finger action preparation and execution were modeled, with three located in the frontal lobe (motor system, following Garagnani and Pulvermüller [15,16]) and three in the parietal lobe (somatosensory system—see Section 2.2 for more details);
- Within-area connectivity by local excitatory and inhibitory connections (see iv)—excitatory connections were sparse, random, and initially weak, exhibiting a neighborhood bias toward close-by links [101,102], following Garagnani et al. [13];
- Between-area connectivity carefully replicating the known neuroanatomical links existing between homologue brain regions—see Figure 1 and Section 2.1 for more details;
- Uniform white noise in all neurons of all areas during both learning and spontaneous network activity, simulating baseline neuronal firing, following Garagnani and Pulvermüller [15].

### 2.1. Network Structure and Function

The network comprises a “motor” and a “somatosensory” system, each containing three areas (see Figure 1), linked according to converging evidence from several neuroanatomical studies (see below). The three “motor” system areas simulated dorsal primary motor (M1, BA 4), dorsal premotor (PM, BA 6), and dorsal prefrontal (PF, BA 8/9/46) cortices. The “somatosensory” system areas simulated primary somatosensory (S1, BA 3a/3b/1/2), somatosensory association (SA, BA 5), and posterior parietal association (PA, BA 7) cortices. The motivation for the inclusion of these specific areas is given below (Section 2.2). As in all previous models based on this architecture [13,14,15,16,17,18,19,20,21,22,98] (see Pulvermüller [24] for a recent review), each model area consists of an excitatory and an underlying inhibitory layer of 25 × 25 paired cells, each pair representing a single cortical column made up of clusters of pyramidal cells and interneurons. The base implementation of the computational model is identical to previous publications utilizing LIF cells [21,22,23,98,103,104], but for completeness, it is summarized below (Section 2.3), alongside the novel reward-modulated mechanisms implemented for this study.

The between-area links within the model (see Figure 1) are implemented according to documented anatomical connections between the corresponding cortices of the primate brain. As previously modeled [15,16], the dorsal primary motor, premotor, and prefrontal cortices (together referred here as the “motor” system) have reciprocal neuroanatomical links between each pair of adjacent areas [105,106,107,108,109,110,111,112], with the prefrontal cortex also having documented links with the primary motor cortex [113,114]. The “somatosensory” system areas are similarly interconnected to their adjacent areas and “next-neighbor” areas, as shown in macaques [115,116], and demonstrated with diffusion tractography and effective connectivity methods in humans [117]. White matter fibers “bridging” the two motor and somatosensory systems are also well documented, with the primary motor and premotor areas linked to the primary somatosensory and somatosensory association areas [115,116,117,118,119] and the posterior parietal association area linked to both the premotor and prefrontal areas [116,117,120].

### 2.2. Modeling Approach and Assumptions

We modeled the acquisition of fine hand/finger motor skills in humans (e.g., object grasping for infants, tool-mediated hand actions—e.g., drawing—in later learners). As elaborated below, this process putatively involves the cortical areas within the frontal and parietal lobes identified in Figure 1A, and the presence of a reward signal (see also Section 2.2.1 below).

Specifically, we modeled those areas in the frontal lobe known to be involved in the preparation and execution of hand/finger movements [106,107,121]—labeled M1, PM, and PF in Figure 1A. The dorsolateral primary motor (M1) and adjacent premotor (PM) areas are involved in action commitment [122,123,124] and have been shown to contain hand and finger representations [107,111]. The dorsolateral prefrontal cortex (PF) has been implicated in action selection [125,126,127], such as when faced with competing possible responses [128]. Within the parietal lobe, we identified and modeled relevant areas (referred to as the “somatosensory” system) known to be involved in the processing of haptic and proprioception information during control and co-ordination of hand and finger movement [129,130,131,132,133,134]—labeled S1, SA, and PA in Figure 1. The primary somatosensory area (S1) is arranged somatotopically with identified hand/finger representations [135,136,137] and is involved in the sensory monitoring of grasping and object interaction [138]. S1 has also been shown to be modulated by M1 during action preparation, allowing for the decoding of the planned effector, as similarly predicted by activity in M1 [139,140,141] and by PM during the execution of voluntary movements [142]. The somatosensory association (SA) and posterior parietal association (PA) areas have been proposed as sites for the internal representation of the body’s state by integrating sensory and motor signals [143], with hand/finger representations having been identified in SA [144]. Both SA and PA have been shown to support prehension preparation [145] and the related positioning of the arm and hand while reaching without any visual input [146]. More generally, these two systems have been shown to interact during motor skill learning and retention [147,148,149], with plasticity changes within the somatosensory cortex observed prior to the motor cortex [150] and disruption via TMS to the somatosensory cortex shown to affect both the learning and retention of motor skills [151,152,153].

Based on the above, we hypothesized that, as a result of learning, repeated finger/hand action execution (in the presence of an object) induces the formation of motor-haptic associations in the cortex, cell assembly (CA) circuits consisting of strongly connected neurons distributed across frontal and parietal areas that link up motor patterns in M1, induced by the execution of a hand action, with co-occurring sensory information in S1, produced by the haptic feedback generated by the hand/finger movement. To simulate the emergence of such CA circuits, we subjected the model to a training process replicating that used in previous studies with this architecture, not involving any “reward” signal (i.e., using just Hebbian-like learning mechanisms)—see Section 2.4 for details.

#### 2.2.1. Modeling Reward: Neurobiological Grounding

Following Refs. [154,155], we simulated the extrinsically motivated internal reward as a global “signal”, broadcast to the entire set of neurons in the network whenever a set of specific conditions occurred (detailed in Section 2.4). Specifically, we adapted the existing neurophysiologically grounded Hebbian-like learning mechanism [99,156], used in previous studies utilizing the present neural architecture [24], so that in the presence of the reward signal—modeling a release of dopamine (DA)—the following changes were applied: (i) the magnitude of the weight change, ∆*w*, induced by either LTP or LTD was increased by 50%, and (ii) the threshold, *θ*_+_, required for LTP to occur was reduced by 25% (as defined by Equations (10) and (11), respectively, in Section 2.3, below).

Effect (i) of the simulated reward, namely, a 50% “boost” in the magnitude of the synaptic efficacy change induced by (Hebb-like) learning, is grounded in several experimental results demonstrating increases in LTP magnitude of up to approximately 50%. Specifically, in the presence of DA, not only was LTD converted into LTP under a negative spike pairing protocol, but also, the magnitude of the EPSP change was up to approximately 20% greater after a positive pairing [157] (their Figure 3). In the hippocampus, both genetic deactivation and pharmacological inhibition of D1 receptors caused an overall reduction in the magnitude of the EPSP change of approximately 50% [158] (their Figure 3A’), whereas dopamine agonists increased the magnitude of LTP by up to approximately 25% [159] (their Figure 3). LTP in the rat PFC was enhanced by approximately 35–45% in the hippocampo-PFC pathway when the DA concentration was increased through infusion [160] and ventral tegmental area (VTA) stimulation [161], as well as when a D1 receptor agonist was applied [67]. These increases in LTP may involve the protein kinase A pathway, as dopamine has been shown to promote the synthesis of AMPA receptors in the hippocampus [162] and to enhance AMPA receptor trafficking to the membrane in PFC [163] along timelines that are compatible with the previously mentioned studies.

Effect (ii) of simulated reward (a reduction in the LTP threshold, the level of postsynaptic depolarization needed for synaptic change to involve an increase—LTP—as opposed to a decrease—LTD—for the same level of presynaptic activity) is motivated by experimental evidence showing that an inverted-U-shaped relationship exists between DA and the level of activity required for LTP to occur, where spike trains that produce no change or LTD can be converted into LTP in the presence of DA [59,60,71,157,162,164], and that the population size of tonically active DA neurons plays a role in modulating the learning rate [165]. Note that here, we did not model the upper bounds of DA concentration where excessive dopamine results in no change occurring.

Finally, we should note that, in the context of the brain areas modeled (see Section 2.1), DA has been shown to modulate plasticity in the motor [70,75,166,167], prefrontal [68,160,161], and somatosensory cortices [75,168], receiving dopaminergic projections from the VTA [161,166].

### 2.3. Model Specifics

Each of the 6 simulated areas is implemented as two layers of artificial neuron-like cells, 625 excitatory and 625 inhibitory, resulting in a total of 7500 cells (illustrated in Figure 1B Inset). Each excitatory cell consists of a leaky integrate-and-fire (LIF) neuron with adaptation and simulates a single pyramidal cell, while its twin inhibitory cell is a graded response cell simulating the inhibitory response of the cluster of interneurons situated within the same cortical column [169,170].

In what follows, Equations (1)–(9) and (12) are identical to the model specifics in all previous publications that used this architecture with LIF cells [21,22,23,98,103,104]. The novel mechanism of dopamine-modulated learning is described in Equations (10) and (11).

Each LIF cell *x*’s output is dictated by its membrane potential, *V*(*x*,*t*), at time *t*, calculated according to the following:

(1) $$\tau \cdot \frac{dV\left(x,t\right)}{dt}=-V\left(x,t\right)+k_{1}\left(V_{in}\left(x,t\right)+k_{2}\eta \left(x,t\right)\right)$$

Here, *V_in_*(*x*,*t*) is the net postsynaptic potential input to cell *x* at time *t* (defined below in Equation (2a–e)), *η*(*x*,*t*) is a white noise process uniformly distributed over [−0.5, 0.5], both *k*_1_ and *k*_2_ (set to zero for inhibitory cells) are scaling constants, and *τ* is the time constant for the membrane. In line with all previous simulations employing this architecture [13,14,15,16,17,18,19,20,21,22,23,98,171,172], the presence of inherent noise in each cell’s activity models the spontaneous firing of real cortical neurons. *V_in_*(*x*,*t*) is calculated as follows, where *A* is the area that cell *x* is a member of:

(2a) $$V_{in}\left(x,t\right)=V_{b}$$

(2b) $$\;+k_{ffb}\cdot \sum_{y\notin A}w_{x,y}\cdot \phi \left(y,t\right)$$

(2c) $$\;+k_{rec}\cdot \sum_{y\in A}w_{x,y}\cdot \phi \left(y,t\right)$$

(2d) $$\;-k_{inh}\cdot \sum_{inh\in A}w_{x,inh}\cdot \phi \left(inh,t\right)$$

(2e) $$\;-k_{G}\cdot \omega_{G}\left(A,t\right)$$

Here, $V_{b}$ in Equation (2a) represents a constant baseline input, Equation (2b) is the total weighted output from neighboring areas scaled by *k_ffb_*, Equation (2c) is the total weighted output from within the same area scaled by *k_rec_*, Equation (2d) is the total weighted output from local inhibition scaled by *k_inh_*, and Equation (2e) represents global inhibition for area *A* as defined by Equation (8) and scaled by *k_G_*. Both Equation (2b,c) take into account the synaptic strengths of the connections between excitatory cells by means of the weights established through learning (*w_x,y_*), whereas in Equation (2d), for inhibitory cells, *w_x,inh_*, is set to 1.

The output of the cell, ϕ(x,t), is then derived from the membrane potential, based on whether it is excitatory (LIF) or inhibitory (graded response). Excitatory cells are either firing or not, depending on whether they overcome the fixed threshold, *thresh*, and do not have a specific reset mechanism, as below:

(3) $$\phi \left(x,t\right)=\left\{\;\begin{array}{cc} 1 & if\;V\left(x,t\right)-\varphi (x,t)>thresh\\ 0 & otherwise \end{array}\right.$$

Inhibitory cells, on the other hand, output the following:

(4) $$\phi \left(x,t\right)=\left\{\;\begin{array}{cc} 0 & if\;V(x,t)<0\\ V(x,t) & otherwise \end{array}\right.$$

For excitatory cells, the adjustment of threshold, φ, in Equation (3) follows:

(5) $$\varphi \left(x,t\right)=\alpha \cdot \omega \left(x,t\right),$$

where the approximate time-averaged firing rate, *ω*(*x*,*t*), is multiplied by an adaptation strength *α*—this has the effect of preventing continuous firing after a spike occurs. This time average is estimated as a low-pass filter of cell *x*’s output, ϕ, assuming that the average at *t* = 0 is zero, according to

(6) $$\tau_{A}\cdot \frac{d\omega \left(x,t\right)}{dt}=-\omega \left(x,t\right)+\phi \left(x,t\right)$$

A cell’s estimated instantaneous mean firing rate, *ω_E_*(*x*,*t*), used to specify the network’s Hebbian plasticity rule (see Equation (9) below) under slower temporal dynamics than Equation (6), is defined by

(7) $$\tau_{Favg}\cdot \frac{d\omega_{E}\left(x,t\right)}{dt}=-\omega_{E}\left(x,t\right)+\phi \left(x,t\right)$$

In addition to the local excitatory–inhibitory circuits explained previously, which mediate local competition mechanisms [173,174], the network also implements an area-specific inhibitory mechanism, which primarily keeps the total (“global”) firing activity of excitatory cells in an area within physiological levels [101]. This mechanism is assumed to be slower than the excitatory–inhibitory dynamics and is realized by a single graded-response unit that estimates the total firing activity within a model area and inhibits all excitatory neurons proportionally by the same amount. The area-specific amount of global inhibition, *ω_G_*(*A*,*t*), for area *A* at time *t* is, therefore, defined by

(8) $$\tau_{S}\cdot \frac{d\omega_{G}\left(A,t\right)}{dt}=-\omega_{G}\left(A,t\right)+\sum_{x\in area}\phi \left(x,t\right)$$

This global inhibition is then subtracted as part of calculating *V_in_*(*x*,*t*) in Equation (2e).

The low-pass dynamics of the cells (Equations (1), (3), (4), and (6)–(8)) are integrated using the Euler scheme with step size ∆*t* = 0.5 in arbitrary time units.

Excitatory links within and between (possibly non-adjacent) model areas are established at random and limited to a local topographic neighborhood; weights are initialized independently and at random, uniformly distributed in the interval [0, 0.1]. The probability of a synapse being created between any two cells falls off with their distance [101] according to a Gaussian function clipped to 0 outside the chosen neighborhood (a square of size n = 19 for excitatory and n = 5 for inhibitory cell projections). This produces a sparse, patchy, and topographic connectivity, as typically found in the mammalian cortex [101,102,175,176].

Synaptic plasticity implemented the Artola–Bröcher–Singer (ABS) model of LTP and LTD, grounded in experimental data [99,156], where the weight, *w_t_*(*x*,*y*), at time *t*, dictating the strength of the connection between cells *x* (presynaptic) and *y* (postsynaptic), is updated according to the following:

(9) $$w_{t+1}\left(x,y\right)=\left\{\begin{array}{lll} {\;w}_{t}\left(x,y\right)+∆w & \left(LTP\right) & if\;\omega_{E}\left(x,t\right)\geq \theta_{pre}\;and\;V(y,t)\geq \theta_{+}\\ w_{t}\left(x,y\right)-∆w & \left(LTD\right) & if\;\omega_{E}\left(x,t\right)\geq \theta_{pre}\;and\;\theta_{-}\leq V(y,t)<\theta_{+}\\ w_{t}\left(x,y\right)-∆w & \left(LTD\right) & if\;\omega_{E}\left(x,t\right)<\theta_{pre}\;and\;V(y,t)\geq \theta_{+}\\ w_{t}\left(x,y\right) & \left(no\;change\right) & otherwise \end{array}\right.$$

Here, ∆*w* is the weight change to be applied, as defined by Equation (10), below; *θ_pre_* is the threshold of presynaptic activity necessary for LTP/LTD; and *θ*_−_ and *θ*_+_ are the postsynaptic thresholds—the latter as defined in Equation (11), below.

Previous implementations of this learning rule have statically defined both ∆*w* and *θ*_+_; however, here, they have been extended to instead be modulated by the reward signal, *R_signal_*(*A*,*t*), present in area *A* at time *t*, as previously described in Section 2.2.1. The weight change that is applied (∆*w* in Equation (9)) is now redefined as

∆*w* = ∆*w_base_*·(1 + (∆*w_multi_*·*R_signal_*(*A*,*t*))), (10)

where ∆*w_base_*, ∆*w_multi_* are small (<<1) constants representing the base learning rate to be applied and the additional amount to multiply it by, respectively, and *R_signal_*(*A*,*t*) is the reward signal present in area *A* at time *t*—set to 1.0 for all areas in the presence of reward, and 0.0 otherwise (see Section 2.4). Compatibility with previous implementations is maintained when ∆*w_multi_* = 0 or *R_signal_*(*A*,*t*) = 0.

The LTP threshold (*θ*_+_) is similarly modulated by the reward signal:

*θ*_+_ = *θ*_+*max*_ − ((*θ*_+*max*_ − *θ*_+*min*_)·*R_signal_*(*A*,*t*)) (11)

Here, *θ*_+*min*_ and *θ*_+*max*_ define the range of values that *θ*_+_ can take, and *R_signal_*(*A*,*t*) is the reward signal present in area *A* at time *t*. Compatibility with previous implementations is maintained when *θ*_+*min*_ = *θ*_+*max*_ or *R_signal_*(*A*,*t*) = 0.

The above learning rule (Equation (9)) is only applied when at least one of the pre- and postsynaptic neurons is itself spiking at time *t* (as per Equation (3)), implementing a form of STDP:

*ϕ*(*x*,*t*) = 1 ∨ *ϕ*(*y*,*t*) = 1 (12)

The full set of values used for the parameters is provided in Table 1.

### 2.4. Procedures, Modeling Approach, and Experimental Design

We created 18 distinct networks, each replicating the overall architecture shown in Figure 1B. As the exact set of synaptic links between cells belonging to areas that are connected is determined at random, and because the synaptic weights are also randomly initialized, we took each network to model a single “subject”.

Each network underwent a two-phase learning process, described below:

- *Phase I*: The network was subjected to repeated presentation of 12 different pre-defined pairs of activity patterns to its “primary” areas (M1, S1), with each pattern pair representing a possible finger/hand motor action and corresponding sensory (haptic) feedback. This phase terminated once the network had been confronted with each pattern pair 1000 times (for a total of 12,000 presentations). Pattern-pair presentations were alternated in random order. Full details of this training phase are given in Section 2.4.1 and below.

As a result of (Hebbian-like) learning mechanisms, Phase I led to the emergence, in each network, of 12 distinct, input-specific cell assembly circuits, sets of strongly connected cells spanning the 6 model areas that linked up the “action” and “perception” patterns co-presented to areas M1 and S1 during training. This phenomenon closely replicated results obtained in previous studies utilizing this neural architecture [13,14,15,16,17,18,19,20,21,22,98] (see Pulvermüller [24] for a recent review). Note that Phase I did not involve reward-modulated learning (i.e., *R_signal_*(*A*,*t*) = 0 for each area *A* and for all simulation steps *t*); in other words, the learning mechanisms at work were purely Hebbian-like, associative LTP and LTD (see Equation (9)).

The effects of Phase I—namely, the emergence of 12 CA circuits in the network –created the necessary conditions for the implementation of the second learning phase:

- *Phase II*: The network was let “free” to run, with its activity driven solely by neuronal noise (simulating spontaneous baseline firing). No “sensory” or “motor” input was provided during this phase. Under such conditions, CA circuits spontaneously and repeatedly ignited in seemingly random order. An internal global “reward” signal was provided to the network whenever any of a pre-defined subset of 6 CA circuits ignited. Phase II terminated once a total of 9000 spontaneous CA ignitions (across all 12 circuits) had occurred. Further details about Phase II are provided below (Section 2.4.2).

Phase II also builds upon and extends previous results [15,89,93], which showed that under specific conditions, neuronal noise accumulates and reverberates within CA circuits, leading to their spontaneous, cyclic ignition and “switch off”, brought about by the local and global inhibitory mechanisms. However, while such previous studies used graded response cells, the present architecture successfully reproduced a steady state of regular spontaneous ignitions in a network that used spiking cells, hence providing a novel result.

It is helpful to highlight here what the behavioral correlates of Phases I and II may be. Phase I can be thought of as simulating an infant’s acquisition of an initial, basic repertoire of associations between specific finger/hand actions (carried out on a given object) and corresponding haptic perceptions. More precisely, each “sensorimotor” pattern-pair presentation is taken to model the execution of a specific finger/hand action on a specific object, devoid of any outcome evaluation. The underlying assumption here is that this initial phase is driven entirely by internal motivation, which promotes a random exploration of the space of possible hand actions, in the absence of—or regardless of any—reward signal (see hypothesis H1 in Section 1, Introduction). The simulations replicate this aspect in that the choice of which pattern pair to present next was entirely random.

Phase II, on the other hand, represents the subsequent developmental stage, in which exploration is paired with evaluation in the form of a reward signal (see hypothesis H2); this, in turn, leads to selective reinforcement of just some of the actions executed. Here, reward is modeled as a global “dopamine” signal that modulates the magnitude/effectiveness of the underlying associative learning, promoting actions subjectively experienced as “successful” and gradually leading to the emergence of skilled hand action.

#### 2.4.1. *Phase I*: Standard Network Training (No Reward)

The training of each network followed a standard procedure replicating the same process applied by all previous simulation studies that used this architecture [13,14,15,16,17,18,19,20,21,22,98,171,172] (see Pulvermüller [24] for a recent review); the procedure is reported here for completeness.

First, the network was initialized with random synaptic links with small random weights and then “taught” to associate 12 randomly generated pairs of “motor-haptic” activity patterns, as explained below. Each pair identified two specific sets of 19 active cells in model areas M1 and S1. Each pattern pair was presented a total of 1000 times, with pairs presented in random order. Each presentation involved “clamping” the pre-defined cells in M1 and S1 for 16 simulation steps, simulating down/upstream activity to/from outside the model. This was followed by a variable time period with no stimulation, during which network activity was allowed to return to baseline levels.

As training progressed, 12 input-specific distributed associative CA circuits gradually emerged, each linking up the pair of “sensorimotor” patterns repeatedly presented to M1 and S1. Throughout this learning phase, periodic “snapshots” of the network state, encompassing all existing links between cells and their synaptic weights, were taken.

At the end of Phase I, which we will be referring to as “Pre-reward”, the learning process was temporarily paused, and data about the spontaneous dynamics of the emerged CAs were extracted, while the network weights remained unchanged (i.e., ∆*w_base_* was set to 0.0; see Table 1); details about the data acquisition are provided in Section 2.5. These data allowed us to assess the probability of each CA’s spontaneous ignition and were used to rank all 12 CAs in descending order of ignition likelihood; the resulting ranked list was used in Phase II (as described in Section 2.4.2, below). If two (or more) CAs exhibited the exact same frequency (i.e., probability) of spontaneous ignition, the CA size (number of cells) was used as the tie-breaker, with the larger CA(s) receiving the higher rank.

#### 2.4.2. *Phase II*—Learning via Spontaneous CA Ignitions (with Reward)

In contrast to the training implemented in Phase I (where network activity was driven by random pattern-pair presentations), in this second phase, activity was driven purely by neuronal noise (i.e., no “sensory” or “motor” input patterns were presented). Two identical copies (“twins”) of the network resulting at the end of Phase I were created, and each of them was run independently, under different conditions (both having learning enabled: ∆*w_base_* = 0.001; see Table 1), as explained below.

First, the list of 12 CA circuits ranked in decreasing order of spontaneous ignition probability (obtained at the end of Phase I) was used to split the set of CAs into two disjoint subsets of 6 each (labeled Group A and Group B in what follows); this was achieved by applying a process (see Table 2) that assigned each CA to either of the two groups on the basis of their rank to balance the overall spontaneous CA ignition probability. Then, in one of the two “twin” networks, Group A was assigned to be the “rewarded” subset of circuits (i.e., spontaneous ignition of any of its members would induce the global “reward” signal—the “rewarded” condition, as explained below), while ignitions of CAs in Group B elicited no reward (the “unrewarded” condition). In the second twin network, the reverse conditions were applied (A was unrewarded and B was rewarded). We will refer to these two conditions as “PhaseII_GroupA-Rewarded” and “PhaseII_GroupB-Rewarded”, respectively. This orthogonal design was needed to account for possible differences that might exist between the two groups of 6 CAs and which were not necessarily controlled for by the “rank balancing” algorithm.

During Phase II, the global reward signal was “switched on”; that is, the value *R_signal_*(*A*,*t*) was set to 1.0 in all 6 model areas—see Section 2.3—whenever one of the CAs in the rewarded group ignited and reset to 0 as soon as CA activity fell below the 50% threshold again (the methodology used to recognized the ignition of a CA is illustrated in Section 2.5). Throughout this learning phase, periodic “snapshots” of the network state, encompassing all existing links between cells and their synaptic weights, were taken. Phase II terminated when 9000 spontaneous CA ignitions had occurred in each twin network.

At the end of Phase II, at a time point that, in what follows, will be referred to as “Post-reward”, the learning procedure was paused again, and data about the spontaneous dynamics of the resulting CA circuits were extracted from each network instance from each condition, while weight configuration remained unchanged (see Section 2.5 for details about the data acquisition).

The above orthogonal design facilitated the effects of learning in Phase II on the twelve CA circuits that the network had learned during Phase I to be measured separately for each of the two subsets of the six CA circuits (Group A and Group B) in the two initially identical networks, with the same group being both rewarded in one “twin” and unrewarded in the other. This is explained in Section 2.5 and Section 2.6, below.

### 2.5. Data Acquisition

During Phases I and II, the emerging cell assembly circuits were identified using the operational definition of a CA circuit adopted in all previous publications that used this architecture. In short, the instantaneous firing rate of each (excitatory) cell—defined by *ω_E_*(*x*,*t*) (see Equation (7))—was monitored during input pattern pair presentation (lasting 16 simulation time steps). A cell was considered to be active in response (or “responsive”) to a pattern pair, *w*, if its activity during this period reached a given threshold, defined separately for each input (*w*) and model area (A) as follows:

(13) $$\theta \left(w,A\right)=\gamma \cdot \underset{e\in A}{\max}\left({\omega \left(e,t\right)}_{w}\right),$$

where *ω*(*e*,*t*)*_w_* is the (estimated) time-averaged output of cell *e* at time *t* (Equation (7)) during the presentation of input *w*, and *γ* is a constant between 0 and 1 (typically, *γ* = 0.50). Simply put, if *m* was the excitatory cell maximally responsive to stimulus *w* in area *A*, threshold *θ*(*w,A*) would be 50% of the mean response of cell *m* to stimulus *w*. All cells responsive to input *w* were considered part of the emerging CA circuit specific to that input.

Instantaneous cell-assembly circuit activity was defined as the percentage of active CA cells within the circuit, across all model areas. A CA circuit was thus considered to have *ignited* if (and only if) its activity reached 50%. Finally, an ignition *episode* was considered to have begun when one or more CA circuits crossed the 50% activity threshold and to have ended when all such CAs had ceased their ignitions (their individual activities had all dropped below 50%).

The above definitions have been consistently used in previous studies to identify CA circuits as sets of input-specific cells [13,16,18,19,20,21,22,23,103,172].

Using the above definitions, at the end of both Phase I (Section 2.4.1) and Phase II (Section 2.4.2), data about the spontaneous dynamics of the emerged CAs were extracted from each network instance (one from the “pre-reward” time point and two from the “post-reward” time point of the two twin networks—see Section 2.4.2 and below), while the synaptic weights remained unchanged (i.e., learning was paused by setting ∆*w_base_* to zero; see Equation (10) and Table 1). Specifically, starting from its “pre-reward” state (the end of Phase I), the network was allowed to run in the absence of any input, so the activity was driven entirely by noise, and no learning, until a total of 2000 spontaneous CA ignition episodes had occurred; during this period, per-area within-CA activity was recorded. This produced a dataset consisting of twelve time series from which the overall frequency and duration of the spontaneous ignition of the previously learned twelve CA circuits (Group A and Group B) during the recorded period could be extracted.

The data extraction process described above was also applied to the two (different) networks that resulted from subjecting the two “twin” models (see Section 2.4.2) to the “PhaseII_GroupA-Rewarded” and “PhaseII_GroupB-Rewarded” conditions, at the “post-reward” time (i.e., end of Phase II). Similarly, this produced a dataset for each network (or condition). In sum, three datasets for each “subject” were obtained: one in the Pre-reward condition (gathered at the end of Phase I) and two in the Post-reward condition (gathered at the end of Phase II, one per condition).

Finally, from each of these three network-state “snapshots” (taken at different time points and conditions), CA-circuit sizes were extracted. This was achieved by applying the same method described in Section 2.4.1, in which each pattern pair is presented for a fixed number of steps after a period without stimulation (however, here, learning was suspended, and each stimulus pattern was only presented once to measure cell responsiveness to that input pattern).

### 2.6. Data Analysis

For each of the 18 “subjects”, and for each of the three datasets obtained from each of them (see Section 2.5), the total number of CA “co-activations”—where two or more CAs reach the activity threshold for ignition at the same time—was computed. Using the interquartile range of the number of CA co-activations across all networks, outliers were determined and excluded from the rest of the analysis; this is because a network containing an unusually high number of co-activations could contain a “spurious” reward effect. For example, if a pair of “rewarded” and “unrewarded” CAs repeatedly co-activated during Phase II, this would result in the unintentional strengthening of a circuit that had not been assigned to the rewarded group of CAs. Additionally, to ensure that no confounds due to different CAs interacting with each other would affect the results, in all remaining “subjects”, the analysis excluded any ignition episode containing a co-activation.

The following ignition metrics were then calculated for each of the three datasets in each subject:

- *Ignition frequency*: Computed as the total number of times each individual CA’s activity crossed the 50% threshold (hence, igniting) across the duration of Phase II.
- *Ignition duration*: Calculated as the time steps between a CA reaching the ignition threshold (the ignition start) and the moment when the CA’s activity was below threshold again (the ignition end).

The three datasets obtained from a single network contained information about the spontaneous dynamics of all twelve CA circuits (extracted as described in Section 2.5); however, the two “Post-reward” datasets contained the results of subjecting the two subsets of six CAs (Group A and Group B) to different—symmetric—reward conditions during Phase II (see Section 2.4.2). Thus, the above metrics were applied separately to each of the two CA groups in each dataset, producing a total of 6 data points per metric. Repeated measure ANOVAs were then conducted on the resulting data, with two factors—group (2 levels: A, B) and reward (3 levels: Pre-reward, Rewarded, Unrewarded). Mauchly’s Test of Sphericity was conducted to determine if any adjustments were required to the degrees of freedom of each test. An alpha level of 0.05 was used, and all analyses were performed in MATLAB R2023b [177].

## 3. Results

Of the 18 networks, 3 (#3, #7, and #15) were excluded after their Phase II as representing outliers in terms of excessive CA co-activations (see Table 3).

### 3.1. Cell Assembly Size

**Figure 2:**
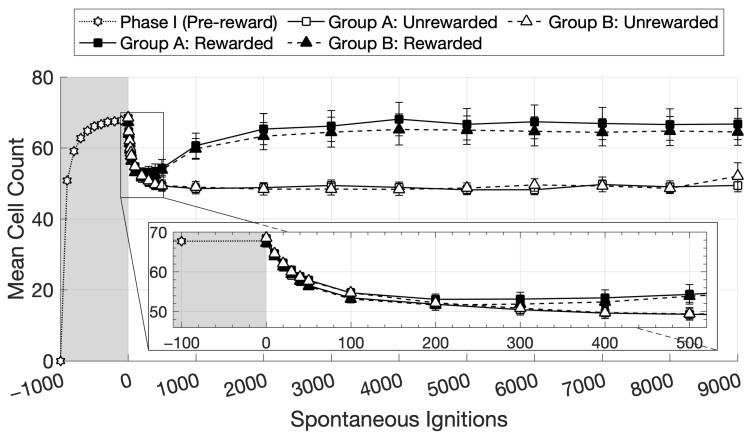
Cell assembly size during learning Phases I and II. Average CA size (total no. of cells) across 15 networks is plotted as a function of the (i) number of pattern-pair stimulus presentations (gray-shaded area, Phase I) and (ii) number of total spontaneous CA ignitions (non-shaded area, Phase II) for the different conditions. Note, in Phase II, the initial drop in CA size between time points 0 and 300 observed across conditions (see inset) and subsequent increase between time points 300 and 1000 present for the CAs in the Rewarded conditions (filled marker curves) but absent in the Unrewarded ones (unfilled markers). Error bars indicate standard error.

Figure 2 plots average CA size across the remaining 15 “subjects” as a function of learning phase, with data for each network collapsed over the 12 CA circuits during Phase I, and over 6 CA circuits within each group in Phase II, whose sizes were assessed under two conditions (Rewarded and Unrewarded). The plots suggest that, as Phase II progressed, rewarded CAs generally became larger than unrewarded ones.

To statistically corroborate this observation, one-tailed paired T-tests were carried out for the 500, 1000, and 2000 time points (Figure 2), collapsing the data across groups for the two conditions. At each of these points, CA size was larger in the Rewarded than in the Unrewarded condition (*p* < 0.01 in all three). Specifically, the t-statistic returned *t*(29) = 4.13 at time point 500, *t*(29) = 6.34 at time 1000, and *t*(29) = 6.65 at 2000. Finally, the CA size of the Rewarded condition at time point 1000 was larger than at time point 500 (*t*(29) = 4.73, *p* < 0.01), confirming a significant increase in the curve.

**Figure 3:**
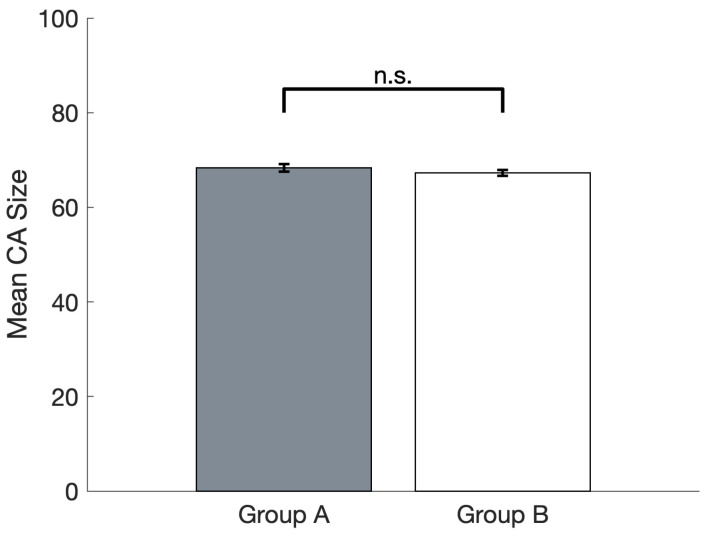
Comparison of Group A and Group B mean CA sizes across all included networks in the Pre-reward condition (end of Phase I). The means of the two groups were not significantly different (*p* = 0.18, n.s.).

A two-tailed paired T-test was carried out to compare the mean CA sizes of groups A and B (Figure 3); this showed that the two Pre-reward means were not significantly different (*t*(14) = 1.41, *p* = 0.18, n.s.).

### 3.2. Ignition Frequency

**Figure 4:**
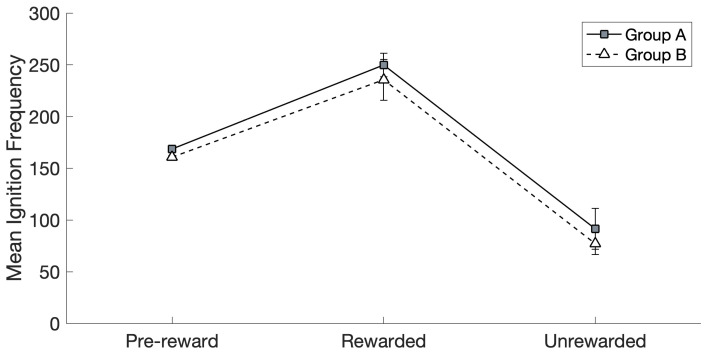
Frequency of spontaneous CA ignition under three different conditions. The mean ignition frequency was significantly higher for Rewarded CAs (end of Phase II) than for the same CAs prior to any learning with reward (Pre-reward condition, end of Phase I), and significantly lower for Unrewarded CAs (end of Phase II) than for the same CAs prior to any reward learning (*p* < 0.01 for both). Error bars indicate standard error (not visible at this scale for the Pre-reward condition).

When investigating the effects of reward and group on ignition frequency (see Figure 4), Mauchly’s test (*χ*^2^(14) = 223.19, *p* < 0.01) revealed that the assumption of sphericity had been violated, and therefore, a Greenhouse–Geisser correction was applied (ε = 0.30) to the degrees of freedom. The thus-corrected repeated measures (RM) ANOVA revealed a significant main effect of reward (*F*(2,28) = 68.64, *p* < 0.01) and no significant main effect of group (*F*(1,14) = 0.46, *p* = 0.51, ns.), and no significant interaction between the two factors was present (*F*(2,28) = 0.07, *p* = 0.79, n.s.).

Post hoc pairwise comparisons (after applying a Bonferroni correction) confirmed that the ignition frequency was significantly higher in the Rewarded than in the Pre-reward (*p* < 0.01) and Unrewarded (*p* < 0.01) conditions (data from Groups A and B were collapsed by virtue of a lack of any significant effect of group, as seen above). The ignition frequency in the Unrewarded condition was significantly lower than in the Pre-reward condition (*p* < 0.01, again collapsing data from the two groups).

### 3.3. Ignition Duration

**Figure 5:**
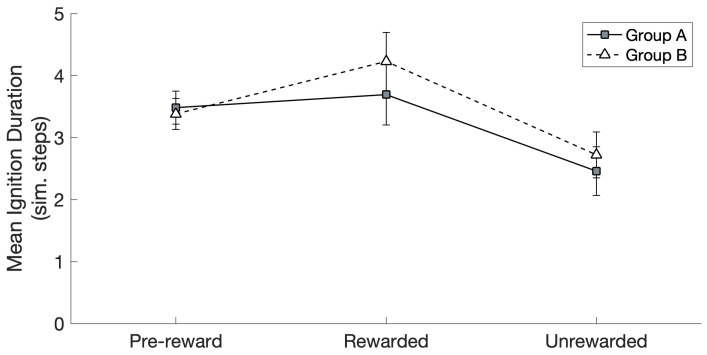
Spontaneous CA ignition duration in three different conditions. The mean ignition duration significantly decreased for Unrewarded CAs as a result of Phase II (learning as driven by spontaneous ignitions, without input stimulus presentation) (*p* < 0.01); Phase II, however, had no significant effect on the ignition duration for Rewarded CAs (*p* = 0.17, ns).

The RM ANOVA carried out on the spontaneous CA ignition duration data (see Figure 5) revealed the significant main effect of reward (*F*(2,28) = 18.69, *p* < 0.01), no significant effect of group (*F*(1,14) = 0.73, *p* = 0.41, ns.), and no significant interaction between the two (*F*(2,28) = 1.53, *p* = 0.23, ns.); again, data were Greenhouse–Geisser-corrected (ε = 0.55). Bonferroni-corrected post hoc comparisons confirmed that the ignition duration was significantly higher in the Rewarded than in the Unrewarded (*p* < 0.01) conditions (data from Groups A and B were collapsed by virtue of the lack of any significant effects of group). There was no significant difference between the Rewarded and Pre-reward conditions (*p* = 0.17, ns.). The ignition duration in the Unrewarded condition was also significantly lower than in the Pre-reward condition (*p* < 0.01).

## 4. Discussion

We used a spiking, six-area deep, brain-constrained model of frontoparietal cortical regions to simulate the natural emergence of reward-seeking behavior in the context of skilled hand action acquisition, and to investigate the neural correlates of reward and the role it plays in early motor development. The noise-driven reverberation and ignition of simulated “motor-haptic” associations, with cell assembly (CA) circuits emerging in the network as a result of learning and repeated “sensorimotor” stimulation, were taken here to represent model correlates of spontaneous decisions to perform a hand action (e.g., to grasp), acquired during an initial exploratory phase in the presence of an object. This was in line with previous computational modeling results [15,89], which had successfully used the same emergent phenomenon to simulate and explain neural correlates of spontaneous decisions to speak and act, albeit without simulating haptic feedback and spiking neurons. We also devised and implemented a novel learning mechanism simulating a global dopaminergic signal, which we used to model the modulatory effects of reward on “classic” LTP/LTD learning; the features of this novel mechanism were grounded in known neurophysiological data about reward-modulated learning in the cortex (see Section 2.2.1).

We found that the subset of CA circuits that were arbitrarily assigned to the group of “to-be-rewarded” actions—representing, for example, particularly successful hand or finger movements—rapidly and spontaneously acquired an advantage in terms of number of spontaneous ignitions over time, over the remaining, “not-to-be-rewarded” ones (see Figure 4). Specifically, at the start of Phase II (the learning stage driven by spontaneous CA ignitions; see Section 2.4.2), Group A and Group B “actions” had the same average probability to spontaneously ignite and the same average size (Figure 3). Note that the specific spontaneous CA ignition probability of each circuit varied within group and network; this is because, due to the network-specific configuration of randomly initialized synaptic links, a result of the Phase I training is that some input pattern pairs happened to induce the emergence of larger—or smaller—CAs, resulting in 12 memory circuits of different sizes and, thus, spontaneous ignition probability. However, at the end of Phase II, in which the continual learning was modulated by a global reward signal applied only to half of the CA circuits (Group A or B), the rewarded “action” subset (regardless of whether it was A or B) ended up with a larger likelihood to ignite than the unrewarded subset. In other words, the system gradually acquired a “bias”, a natural preference for spontaneously attempting “actions” that resulted in a reward. This can be seen as equivalent to the spontaneous development of reward-seeking behavior in an infant or other agent.

We think that the mechanisms underlying the gradual emergence of a reward-driven spontaneous action-decision “policy” in the network, a phenomenon that seemingly developed over about 1000 CA ignitions across the 12 CA circuits (see Figure 2), reside in the effects of the global reward signal on learning. In fact, as can be seen from Figure 2, after an initial drop in the CA cell count in Phase II, circuit size increased to around 65–70 cells per CA for the “rewarding” subset of CAs, but not for the others (whose size remained around 50 CA cells per circuit). We submit that this size increase, promoted by the learning enhancement that the reward signal brought about, was reflected, dynamically, in the significant increase in the probability of spontaneous CA ignition (Figure 4): in fact, in the presence of uniform neuronal noise feeding equally into all circuits, larger assemblies will exhibit a higher likelihood to spontaneously ignite than smaller ones, as more cells imply (i) larger *noise-driven activity* per circuit (each excitatory cell generates the same amount of noise—see Equation (1); hence, *n* cells produce *n* times more “baseline firing” activity than 1 cell) and (ii) a larger number of *synaptic links* per circuit and thus more opportunities for noise to start reverberating in them. This is corroborated by the result that, when CA size across the two groups did not differ (i.e., at the pre-reward time), Groups A and B exhibited an equal probability to spontaneously ignite (see Figure 3 and Figure 4), confirming our initial hypothesis. A second effect of the CA-size increase we observed was an increase in the duration of CA ignition (Figure 5), i.e., of the time during which activity continues to reverberate within a memory circuit (a model correlate of working memory; see also Pulvermüller and Garagnani [17]), as has been previously demonstrated in the presence of additional links between model areas [18].

As previously mentioned, at the start of Phase II, we observed an initial drop in CA size, prior to the point where the curves diverged (see Figure 2, between 0 and 500 spontaneous ignitions). As the CA cell counts in both phases were measured using the same method (see Section 2.4.1 for details), we suggest that this drop might relate to the difference in the activity driving CA ignition between the two phases. In fact, in Phase I, CA ignition is driven by a pre-determined set of 19 cells being “clamped” in areas M1 and S1 for a significant number of steps (16), whereas in Phase II, this is driven by the spontaneous process of noise reverberation within the circuit. We conjecture that, overall, such spontaneous ignitions induce lower levels of activity in the circuit (particularly in areas M1/S1), with the consequent gradual weakening of the links between CA cells (and, hence, an overall reduction in CA size, as circuit size stabilizes around the new level of activity). This is supported by the results plotted in Figure 2: in fact, if overall levels of activity within the CA circuits differ between Phases II and I (due to external stimulation having stopped), this sudden shift in network conditions should manifest as an abrupt, discontinuous transition in the dynamics of CA size evolution at around the 0 time point. This is what can be seen in Figure 2 (inset), which shows a discontinuity—more precisely, a “cusp”—in the curves: a “step” change in the derivative, confirming that the new stimulation conditions introduced immediately affected the overall trend of CA size.

A second effect of the “spontaneous learning” phase (Phase II) was to induce a shorter duration of CA ignition (Figure 5) for the unrewarded condition when compared to the rewarded condition; that is, activity within non-rewarded memory circuits reverberated for a shorter time than within rewarded ones. Analysis of these data, however, revealed no significant difference between the rewarded and pre-reward conditions. We conjecture that this somewhat unexpected result is likely due to the differences in how the circuits are activated during their respective learning phases. In fact, the network’s behavior in the pre-reward condition is measured at the end of Phase I, in which training was carried out via pattern-pair presentation, where a fixed number of cells were activated in the two “primary” (M1 and S1) model input areas for 1000 per-pattern presentations (see above). In the second, reward-driven learning phase (Phase II), instead, cells in M1 and S1 received no external input and only became active as a result of a CA’s spontaneous ignition. Thus, although in Phase II learning was enhanced for the rewarded CAs when they spontaneously ignited, this effect appears to be canceled out by the absence of an external input, which in Phase I conveyed strong activity (for several time steps) as input to 19 cells in each of areas M1 and S1. This hypothesis is supported by the mean duration in the unrewarded condition being significantly lower than the pre-reward condition (as well as the rewarded condition): as CA learning in both pre-reward and unrewarded conditions does not involve any reward signal, the one key difference is the absence of the input signal. The prediction emerging from these results, therefore, is that the reward signal, besides reinforcing the set of most relevant action-related memory circuits and making them more likely to be executed, also acts—indirectly—on the set of “uninteresting” (unrewarded) CA circuits, by making their spontaneous persistence in working memory shorter (the reverberant CA activity duration in the “unrewarded” condition at the end of Phase II is shorter than at the end of Phase I, a phenomenon seemingly related to their reduction in size—see Figure 2). This unanticipated prediction awaits validation by means of experimental testing.

If—as we hypothesized—a spontaneous CA ignition models the neural correlates of an endogenous, “free” decision to act, such a spontaneous decision may not be driven by activity in the primary somatosensory cortex (S1), reflecting the sensory stimulation induced by the presence of an object, as a “free” decision would no longer be such if it was triggered by the perception of a sensory stimulus. (This simulated perception, in turn, would cause the reactivation of the input-specific CA circuit and thus prompt execution of the associated action—e.g., a power grip, or a precision one). In such a situation, one might ask what the behavioral and brain correlates of such spontaneous CA circuit ignition may be: if this phenomenon indeed represents a spontaneous hand action decision, would such a situation simulate an infant attempting to carry out a hand action in the “void”, i.e., without a target object? And, assuming that to be the case, what does the activity that a CA circuit’s ignition elicits in S1 represent? (As previous studies using this architecture have shown [15,89], the spontaneous ignition of a CA that emerged as a memory circuit binding two patterns repeatedly co-occurring in two model areas—here, M1 and S1—partly reconstructs such associated patterns; thus, area S1 would be reactivated by the ignition of a distributed “motor-haptic” circuit. However, as there is no object present, activity in S1 cannot model the sensory/haptic feedback that such an object would induce).

We submit that the activity pattern that the spontaneous “motor-haptic” CA-circuit ignition induces in model area S1 (associated with a specific “action” in M1) represents the neural correlate of the corollary discharge [178,179,180]. Note that this term is often used synonymously with “efference copy” [181]; however, here, we adopt the conceptual distinction suggested by Ford and Mathalon [181]—see their Figure 1 for a summary—where “corollary discharge” refers to the representation of the action’s predicted outcome in the receiving areas (i.e., model area S1′s activity in question here), while “efference copy” refers to the *transmission* of the motor plan to these receiving areas. There is a wealth of evidence in support of the corollary discharge phenomenon [182,183], and it has been suggested as an important mechanism that enables the brain to distinguish between self- and externally generated actions [178] and predict the sensory outcome of a motor plan [178,184]. Its failure has been implicated in conditions such as psychosis and schizophrenia [178,181,185,186,187], for example, where it has been suggested that internal signals are not recognized as such and result in delusions [178] or hallucinations [185]. Thus, one prediction emerging from the model—namely, that activity should be observed in area S1 as a result of a hand action (such as grasping) even in the absence of a target object—is indeed supported by experimental findings, where the motor system has been shown to transmit information regarding future action to primary somatosensory cortex before any sensory feedback has been received [141,188] and to modulate activity in such an area [140,142].

While our model utilizes random exploration and associative learning within a biologically constrained framework, it shares conceptual commonalities with the “action quantization” literature often found in robotics and reinforcement learning, which aims to discretize infinite, continuous action spaces into a finite set of “meaningful” policies or primitives [189,190,191,192]. Much like our initial phase of exploration, quantization approaches often rely on unsupervised or intrinsically motivated objectives, such as entropy maximization [189,193], to ensure broad coverage of the motor space. Quantization methods often treat these primitives as discrete “macro-actions” to be assembled by a high-level reinforcement learning agent for task completion [189,192]. Instead, our model focuses on the reward-based strengthening of specific cell assemblies, thereby uncovering their underlying dynamics. Furthermore, whereas action quantization is primarily a computational strategy for simplifying decision-making, our work emphasizes the biological constraints and neural mechanisms that allow such motor behaviors to emerge naturally within a physiological architecture.

Finally, the brain-constrained approach adopted here requires that the model include relevant cortical areas and their associated links, which, taken together with the implemented neurophysiological constraints (see points i–viii at the beginning of the Materials and Methods section), result in a reasonably complex model. However, while this gives the appearance of a less parsimonious or efficient model, a brain-constrained approach allows us to simulate, understand, and make claims about what may be happening in the brain. Several previous works with this architecture [15,16,18,19,22,23,172] have explored the issue of removing—or adding—model components to elucidate their role in achieving the observed results. While an ablation or full parameter-space exploration falls outside the present scope, we should mention that the emergence of the main phenomena upon which this study relies—namely, the formation of distributed associative memory circuits and their spontaneous ignition—is robust to changes in both the number of network areas and, generally, the type of between-area connectivity—see Pulvermüller [24] for a review.

### 4.1. Model Limitations

The neurocomputational model that we used here, although built explicitly to reflect structural and functional features of the mammalian cortex (see Section 2), implements several simplifications and assumptions. It is also appropriate to repeat here that we are not explicitly modeling the environment in which a cognitive agent acts: the model consists of a closed, “circular” system, in which sensory consequences and evaluation of action success are not derived from external outcomes but simply taken as a given.

One of the main simplifications is perhaps that the reward signal applied was discrete (i.e., either “on” or “off”), based on whether any of the “rewarded” CAs were, respectively, above or below the 50% activity threshold (see Section 2.2.1). However, the real dynamics of dopamine signaling are much more complex, as the rates of release and reuptake affect its overall concentration [194,195,196,197]. Additionally, the distribution of dopamine projections and receptors may not be consistent across the cortex [75,198]. We argue that these simplifications are acceptable, as the main result of this study (i.e., the selective increase in CA size—and, thus, in spontaneous ignition probability—induced by the selective reward signal) should still emerge—albeit possibly with a different effect size—even under different amounts of simulated dopamine, which could also result from differences in the cortical distribution of dopaminergic projections and receptors (see also Section 4.2).

We have assumed here that the reward signal co-occurs with a rewarded action being executed and is, therefore, applied immediately, as in response to the success of the action [12]. As such, we did not attempt to model how dopamine plays its modulatory role at relevant synapses when it is not released until several seconds after the initiating action responsible for it, as is typical of extrinsic reward [50,199]—termed the “distal reward” [200] or “credit assignment” [54] problem. Several models have successfully addressed this by using eligibility traces [201,202], where a synapse is “marked” with a decaying signal so a later-occurring third factor can trigger learning [82,203] or descriptive algorithms such as temporal difference learning [54,204,205], whose error term has been shown to be comparable to the phasic dopamine signal [51]. We submit that this is an acceptable simplification, as the purpose of this study was to investigate *how* the reward signal may affect the development of spontaneous action decisions, rather than any effects relating to *when* it may arrive. The “how” was an important foundation to have established, arguably as a dependency, as we can now build on it further to tackle questions related to the “when”—see Section 4.2 for further discussion.

Finally, the dorsal and ventral streams of visual information processing [206,207] are well known to be involved in the visually guided grasping of objects; specifically, the dorsal stream is believed to support the processing of information relating to the visual co-ordination of grasping and the spatial properties of the object to be grasped, while the ventral one is related to the visual identification of objects [206,207]. These aspects of hand-related action were not modeled here; we argue that this simplification is justified in the context of this proof-of-concept study, aimed at testing whether neural mechanisms underlying reward modulation might lead to the natural emergence of a bias in the spontaneous action behavior of a system. While these cortical areas certainly play a role in the context of skilled hand action learning in infants, from a modeling point of view the inclusion of additional areas to simulate the visual modality, per se, would not change the rationale of the simulations, nor the main result: as already showed in a number of previous simulation studies with this architecture, the introduction of further areas (up to 12—see Refs [14,16,19,20,21,22,23,98,103,104]) left the key phenomenon (namely, the formation of cell assembly circuits distributed across the network) upon which the present study builds unchanged. This modeling decision is also justified on the basis of evidence that infants are able to co-ordinate their actions without visibility of the hand [208], as might be expected during early development, where self-grasping occurs [7,8]. That said, the extension of the model with a visual system would enable simulating, for example, the presence of multiple action affordances [209,210] for the same given (visual) object (modeled as two or more “motor-haptic” CA circuits associated with the same activity pattern present in primary visual cortex, simulating, e.g., the presence of different types of grips for the same object). This important direction for future work is elaborated in Section 4.2 below, along with other possible architectural and methodological extensions.

### 4.2. Future Work

There are three aspects of this specific investigation that could be explored in more detail in the future as valuable extensions of this study. Firstly, it would be useful to understand the relative contribution of the different components of our reward-modulated learning rule (the LTP threshold modulation and the “boosted” synaptic weight increase) to the changes observed in CA size and ignition metrics. Future simulation work could investigate these aspects, in particular to determine if increases in the CA size can be dissociated from increases in ignition frequency or if both components of the learning rule are necessary and the two outcomes are correlated. Secondly, it would be interesting to investigate if there are any differences in which specific cells are recruited and where they are located—in particular, if there is a shift from the input model areas (i.e., M1/S1) toward the more central and densely connected areas, as these input areas are no longer receiving direct input when spontaneously igniting during Phase II. It would also be interesting to understand if the specific cell members changed over time, even if the number of cells within a given area remains consistent, or if, once the CA size has stabilized (as indicated by Figure 2, approximately 2500 spontaneous ignitions), the “location” of the member cells is also “fixed”. Lastly, the relationship between particular quantitative network parameters, such as the number of model neurons and the number of CA circuits, and reward would be an interesting line of investigation to explore effects on overall network capacity. Given that rewarded CAs become larger (Figure 2), this might suggest that having “too many” rewarded CAs would reduce the total number of CAs that a network can support.

Whereas in the present model we have chosen to exclude areas relating to the dorsal and ventral visual streams [206,207], in the context of modeling action development, it would be appropriate to extend the model with one or both of these visual streams to simulate both the grasping of different objects and grasping the same object in multiple ways, as one might expect to occur during skilled hand action development. The latter, in particular, would be an important future direction in the context of CA circuits in order to understand how circuits that overlap to some degree interact, as expected to emerge when two or more “motor-haptic” CA circuits are repeatedly associated with the same “visual” pattern. Previous simulation results [211] have shown that CA circuits compete with each other and that overlapping circuits (with an overlap up to 33%) can develop and co-exist [23]. A direct extension of these modeling works would enable applying this neural architecture to explore neural correlates of alternative forced choices, whereby competing action-related CA circuits linked to the same visual object enter a transient competition before a spontaneous decision to act is reached.

Within the model, we have treated the reward signal as a static value, where, instead, a more realistic implementation should model the dynamics of dopamine more closely [194,195,196,197] and may necessitate further extending the architecture to differentiate between the tonic and phasic dopamine signals [212,213,214], which are suggested to have different functional roles [194]. It would also follow to support a more complete picture of the observed inverted-U effect of dopamine on LTP/LTD [59,60,71,164], further extending the learning rule to model excessive dopamine, which has been demonstrated to cause either LTD or no change to occur [61,62,215], and to model the observed inverted-U-shaped effect on LTD induction [71] in addition to the present implemented effect on LTP.

In order to explore the distal reward problem, this architecture could be extended to enable eligibility traces, as evidence of their existence has been demonstrated in both the cortex [216] and striatum [217]. One possible neurobiological mechanism for the eligibility trace is that of reverberant activity [218], as sustained single-neuron activity has been shown to carry information about outcomes across the 4–6 s between trials [219]. Reverberant activity is a significant feature of the CA circuits that emerge in this architecture, presenting an opportunity to investigate the role it may play in distal reward.

In terms of the simulation approach, the initial pattern-presentation phase of training the model was implemented under the assumption that the motor action and subsequent somatosensory feedback would completely co-occur, whereas, to take a more realistic approach, one would expect the somatosensory feedback to reach the cortex slightly later, once the action has been initiated. Similarly, after a spontaneous action attempt (CA ignition), it is reasonable to assume that proprioceptive input (representing the position of joints and muscles) would also reach area S1, along with whatever somatosensory information the action induces. Extending the simulation approach to incorporate receiving a “response”—in the form of a later stimulus to model area S1—to a spontaneous action would provide interesting opportunities to explore more realistic sensory experiences, such as if a previously learnt action produced a novel sensation [220].

If the model were to be extended to receive such a “response” to a spontaneous action, this would provide an additional means to explore the distal reward problem. While we could plausibly assume that the reward signal co-occurs with such somatosensory feedback activity (such as simulated skin contact induced by grasping), that may not be true of the initiating “motor” activity. The network activity that results from a “response” being received in the model S1 area could be investigated to further understand the role that the links between the somatosensory/parietal and motor areas play, in addition to our suggested “corollary discharge” effect (see Section 4). In particular, whether activity would propagate from the somatosensory areas to the motor areas sufficiently to initiate a “replay” of the original action that co-occurs with the reward signal and can therefore receive reinforcement. This “replay” may also be enabled or enhanced by reverberant activity within motor CA circuits, representing their persistence in working memory, if they were sustained until the arrival of the later-occurring somatosensory activity and subsequent reward signal.

The previously discussed sensory consequences of actions could be explored by tackling the present work’s lack of embodiment, which was adopted because the main focus here was on using a brain-constrained neurocomputational model to identify a neuromechanistic account of the spontaneous emergence of a reward-seeking behavior in infants (and other cognitive agents). The model could, therefore, be incorporated into a physical or simulated agent with the means to interact with an environment through fine motor movement, receive feedback on its actions (such as the somatosensory information from interacting with an object), and evaluate action success based on external outcomes (such as grasping an object). This would provide a platform for more realistic investigations of skilled hand action acquisition and motor control. Within the space of motor control, for example, the use of reinforcement learning has led to the development of various methods to improve motor control algorithms, such as with actor–critic approaches and state-filtered disturbance rejection control [221,222]. As an embodied agent has the opportunity to operate on a continuous action space, it would be interesting to investigate how the present approach’s random sampling of the action space performs when compared to principles from action quantization, which reduce the size of the action space [189,190,191,192]. In this context, future simulation studies with brain-constrained models such as the one adopted here could shed light on the neural correlates of these optimization strategies.

Finally, here we have focused on *extrinsically* motivated action behavior, in which reward is produced by biologically salient action outcomes, such as the attainment of food. However, we also suggest that our novel dopamine-modulated Hebbian learning rule has future potential for modeling the acquisition and consolidation of behaviors driven by *intrinsic* motivations. In this respect, it has been proposed that the initial phase of environmental exploration—during which infants acquire a repertoire of actions, including object grasping and other forms of manipulation—can be driven by intrinsic motivations: mechanisms through which infants gradually form and refine motor skills because of their inherent capacity to affect the environment and to produce novel or surprising outcomes [223,224,225,226,227]. In contrast to extrinsic motivations, which in animals are directed toward obtaining external resources that satisfy biological needs such as hunger, thirst, or pain avoidance [228], intrinsic motivations can be seen as processes oriented toward the acquisition of knowledge and skills that are only later employed to achieve useful outcomes [229,230,231,232], such as during early motor and vocal development [233,234]. Intrinsic motivations are not limited to infants: adults have been shown to organize their explorations based on task difficulty and novelty [235], to prefer novel images over familiar ones [236], and to exhibit enhanced memory performance when intrinsically motivated [237,238,239].

In regard to this future extension, we note that the neuroscientific underpinnings of intrinsic motivations have not been as extensively investigated as extrinsic motivations [240]. However, they may both be facilitated through the same biological processes [223,241]. Since dopamine’s discovery as a neurotransmitter in the brain [242], it has been the focus of a vast body of research revealing its association with reward and reward-related processes [243,244,245], and more recently, it has been suggested to play an important role in intrinsic motivation [204,238,240,246,247]. For example, activity in the striatum, which contains a high density of dopamine receptors [248] and is involved in both reward-related [249] and decision-making processes [250], has been associated with intrinsically motivated task performance [251] and the integration of intrinsic and extrinsic reward value [252] and has been correlated with curiosity [253]. Limited direct evidence specifically linking dopamine with intrinsic motivations also exists through the study of flow states, where individuals enter an effortless and focused “zone” while carrying out an activity that they find inherently enjoyable [254,255]. Subjects who were most prone to flow states were shown to have greater dopamine D2-receptor availability in the striatum [256,257], which has been suggested to mean that the availability of this receptor is implicated in the capacity for intrinsic motivation [240].

## 5. Conclusions

We built and applied a spiking, six-area deep brain-constrained neural architecture simulating the structure and function of frontoparietal areas of the human brain to try to shed light on the neural mechanisms underlying early stages of skilled hand action acquisition in infants. The model incorporated a global reward signal that modulated “classical” Hebbian-like learning mechanisms, simulating dopamine-mediated action reinforcement. We hypothesized that the regular, noise-driven spontaneous ignitions of distributed “action-perception” cell assembly circuits (which emerged during the initial training that simulated motor babbling) can be taken to model neural correlates of spontaneous hand action decisions. On this basis, our results show how a fully brain-constrained, spiking, deep neural network can autonomously develop a “preference” for reward-inducing “hand actions”, purely as a result of noise (simulating baseline firing) and dopamine-modulated associative Hebb-like learning. This manifested as the rewarded memory circuits becoming “stronger”—and, hence, spontaneously reactivating more frequently and for longer durations—than the unrewarded ones. We submit that the present model offers a neuromechanistic account, at the cortical-circuit level, of the natural emergence of reward-seeking behavior as typically observed in infants (and other agents) during development, where dopamine increases the probability that a rewarding action occurs again in the future.

## Figures and Tables

**Table 1:** Model parameters during training (*Phase I*) and reward-modulated learning (*Phase II*).

Parameter	Value
τ (Excitatory)	2.5
τ (Inhibitory)	5
*τ_S_* (Slow)	12.0
*k*_1_	0.01
*k*_2_ (Excitatory)	*N*√48
*N*	15
*k*_2_ (Inhibitory)	0
*V_b_*	0
*k_ffb_*	500
*k_rec_*	500
*k_inh_*	500
*k_G_*	95
*thresh*	0.18
*α*	7.0
*τ_A_*	10.0
$\tau_{Favg}$	30.0
Learning rate base (∆*w_base_*)	0.001
Learning rate multiplier (∆*w_multi_*), *Phase I*	0
Learning rate multiplier (∆*w_multi_*), *Phase II*	0.5
*θ_pre_*	0.05
LTD Thresh. (*θ*_−_)	0.14
LTP Thresh. Min. (*θ*_+*min*_)	0.15
LTP Thresh. Max. (*θ*_+*max*_)	0.20
∆*t*	0.5
*Amplitude* (M1, S1), *Phase I*	500
*Amplitude* (M1, S1), *Phase II*	0

**Table 2:** The process used to assign CA circuits to a subset group on the basis of their ignition probability rank. This aims at balancing Groups A and B in terms of the overall probability of CA spontaneous ignition so that, over a sufficiently long period of time, the total number of ignitions of Group A’s circuits would tend to equal that of Group B’s circuits. In fact, as can be seen from the table, after assigning the CA igniting most frequently (i.e., with rank #1) to Group A, the next CA down (rank #2) must be allocated to the other group (Group B). However, as this necessarily creates an initial “bias” toward Group A (the top-ranked CA likely has a larger frequency—or probability—of spontaneous ignition than the second), the next two CAs down (ranks #3 and #4) should be assigned in “reversed” order, i.e., to Group B and Group A, respectively. The same line of reasoning is then applied to assign CAs with ranks #5–#8 but starting from Group B (once again in the attempt to offset the initial bias). The process is then repeated for the bottom-four ranked CAs (#9–#12).

CA Rank #	Group A	Group B
1	✓	-
2	-	✓
3	-	✓
4	✓	-
5	-	✓
6	✓	-
7	✓	-
8	-	✓
9	✓	-
10	-	✓
11	-	✓
12	✓	-

**Table 3:** Percentage of CA co-activations per network and reward group, with excluded outliers highlighted in bold.

Network	Pre-Reward	Group 1	Group 2
1	0.65%	0.90%	2.50%
2	0.75%	2.45%	2.45%
**3**	**21.35%**	**52** **.** **25%**	**47** **.** **15%**
4	1.30%	2.30%	1.95%
5	0.20%	0.55%	0.30%
6	2.65%	4.85%	3.30%
**7**	**1.10%**	**1** **.** **20%**	**47** **.** **90%**
8	0.60%	1.50%	1.75%
9	3.10%	2.85%	2.35%
10	0.80%	1.50%	1.55%
11	3.80%	5.45%	4.20%
12	0.75%	0.45%	1.10%
13	0.05%	0.00%	0.00%
14	0.40%	0.95%	0.95%
**15**	**2.35%**	**38** **.** **70%**	**3** **.** **70%**
16	0.90%	2.20%	2.60%
17	0.20%	0.80%	0.60%
18	1.05%	1.20%	2.00%

## Data Availability

The original data presented in the study are openly available in OSF at https://osf.io/6fqvw/overview (accesed on 21 January 2026).
